# Amorphous calcium phosphate and its application in dentistry

**DOI:** 10.1186/1752-153X-5-40

**Published:** 2011-07-08

**Authors:** Jie Zhao, Yu Liu, Wei-bin Sun, Hai Zhang

**Affiliations:** 1 Stomatological Hospital, Nanjing University Medical School, 30 Zhongyang Road, Nanjing 210008, China; 2 School of Dentistry, University of Washington, 1959 NE Pacific St, Seattle, WA 98195-7456, USA

## Abstract

Amorphous Calcium Phosphate (ACP) is an essential mineral phase formed in mineralized tissues and the first commercial product as artificial hydroxyapatite. ACP is unique among all forms of calcium phosphates in that it lacks long-range, periodic atomic scale order of crystalline calcium phosphates. The X-ray diffraction pattern is broad and diffuse with a maximum at 25 degree 2 theta, and no other different features compared with well-crystallized hydroxyapatite. Under electron microscopy, its morphological form is shown as small spheroidal particles in the scale of tenths nanometer. In aqueous media, ACP is easily transformed into crystalline phases such as octacalcium phosphate and apatite due to the growing of microcrystalline. It has been demonstrated that ACP has better osteoconductivity and biodegradability than tricalcium phosphate and hydroxyapatite in vivo. Moreover, it can increase alkaline phosphatase activities of mesoblasts, enhance cell proliferation and promote cell adhesion. The unique role of ACP during the formation of mineralized tissues makes it a promising candidate material for tissue repair and regeneration. ACP may also be a potential remineralizing agent in dental applications. Recently developed ACP-filled bioactive composites are believed to be effective anti-demineralizing/remineralizing agents for the preservation and repair of tooth structures. This review provides an overview of the development, structure, chemical composition, morphological characterization, phase transformation and biomedical application of ACP in dentistry.

## 1. Review

Amorphous calcium phosphate (ACP) is the initial solid phase that precipitates from a highly supersaturated calcium phosphate solution, and can convert readily to stable crystalline phases such as octacalcium phosphate or apatitic products. Its morphological form, structural model and X-ray diffraction patterns are typical for noncrystalline substances with short-range periodic regularity. ACP has been demonstrated to have better in vivo osteoconductivity than hydroxyapatite (HAP), better biodegradability than tricalcium phosphate, good bioactivity but no cytotoxicity [1]. These excellent biological properties make ACP widely used in dentistry, orthopaedics and medicine. This review provides an overview of the development, structure, composition and morphology characterization, phase transformation and biomedical application of ACP in dentistry.

## 2. Development

Generally, it is believed that ACP was firstly described by Aaron S. Posner [1] in the mid 1960s. It was obtained as an amorphous precipitate by accident when mixing high concentrations (30 mM) of calcium chloride and sodium acid phosphate (20 mM) in buffer [2]. In X-ray diffraction, it was shown to have only two broad and diffuse peaks, with maximum at 25° 2θ. No other features were obvious and it was clearly not apatite. This pattern is typical for substances that lack long range periodic regularity. It was found that immediately after being mixed, the spontaneously formed precipitate was a non-crystalline, or amorphous, calcium phosphate with calcium to phosphorus molar ratio (Ca/P) of 1.50. After several hours, it could convert to poorly crystalline apatite on ageing. Afterwards, this solid converts slowly to crystalline apatite (Ca/P = 1.67) by an autocatalytic mechanism [3].

In 1965, Eanes et al. identified ACP as a bone component [2]. ACP in bone, along with the apatite, might account for the broad diffraction pattern and variable composition of bone minerals. An age-dependent change in the ACP content of bone was also described, with the proportion of ACP decreasing with age [3,4]. In 1975, ACP was found in the mineralized cytoplasmic structure isolated from the blue crab hepatopancreas, with a very similar short-range atomic structure to synthetic amorphous calcium phosphate [5].

## 3. Structure

After the discovery of amorphous calcium phosphate, the early studies were focused on the structure of ACP. It was suggested that synthetic ACP particles, which appear as 300- 1000 Å spheres in the electron microscope, consist of a random assembly of ion clusters 9.5 Å in diameter, dimensions consistent with the chemical composition of Ca_9_(PO_4_)_6 _[5]. And the 15-20% of water found in synthetic amorphous calcium phosphate was shown to be mostly in the interstices between, and not within, the individual Ca_9_(PO_4_)_6 _clusters [6]. Aggregated ACP particles readily dissolve and crystallize to form apatite, a thermodynamically stable phase. The typical radial distribution of noncrystalline ACP cluster structures, calculated from the x-ray diffraction patterns, is only two broad and diffuse peaks showing the rapid drop-off of atomic periodicity. Short-range order exists in these amorphous structures but no long-range order such as that in crystalline hydroxyapatite [6]. Infrared analysis showed a similar lack of crystalline order about the PO_4 _anions in the ACP structure [7].

It is now generally agreed that, both in vitro and in vivo, precipitation reactions at sufficiently high supersaturation and pH result in the initial formation of an amorphous calcium phosphate with a molar calcium/phosphate ratio of about 1.5, with a range of 1.34-1.50 in different pH and 1.50-1.67 when adding different amount of carbonates [8]. However, Wuthier et al reported that ACP, with Ca/PO_4 _molar ratio as low as 1.15, precipitated at more acidic preparative pHs, i.e.6.9 [9].

More importantly, it has been shown that ACP particles are nanometer particles. Primary particle sizes of ACP is about 40-100 nm. The morphology of ACP solids appears to be a curvilinear shape when viewed by TEM, rather than the faceted, angular shape of crystalline calcium phosphates. However, this curvilinear appearance has only been clearly established with dried ACP [10]. The initial flocculates collected immediately after precipitation of highly hydrated ACP have a low-contrast disk-shaped appearance. High-contrast spherical particles begin to appear as ACP suspensions age, and become the dominant shape with time [11].

The disordered structure makes ACP highly reactive with body fluid, resulting fast apatite reprecipitation. Accordingly, ACP has been evidenced to have better in vivo osteoconductivity than hydroxyapatite and better biodegradability than tricalcium phosphate [10]. The ACP precipitate, with little long-range order, is a highly unstable phase and hydrolyzes almost instantaneously to more stable phases. In the presence of other ions or under in vivo conditions, ACP may persist for appreciable periods due to kinetic stabilization [12]. Although the exact mechanism of stabilization of ACP is not understood, the presence of Mg^2+^, F^-^, carbonate, pyrophosphate, diphosphonates, or polyphosphorylated metabolites or nucleotides, in sufficient quantity will prevent the transformation of synthetic ACP to hydroxyapatite [13,14].

## 4. ACP in Biomineralization

It has been stated that ACP likely plays a special role as a precursor to bioapatite and as a transient phase in biomineralization [15]. In solutions, ACP is readily converted to stable crystalline phases such as octacalcium phosphate or apatitic products. One biomineralization strategy that has received significant attention in recent years is mineralization via transient precursor phases [16]. Transient amorphous mineral phases have been detected in biomineral systems in different phyla of the animal kingdom [17]. ACP has been previously reported in the otoliths of blue sharks and also shown to form as a precursor phase of carbonated hydroxyapatite in chiton teeth [18]. The presence of an abundant ACP phase has also been demonstrated in the newly formed zebrafish fin bony rays [19]. The disordered phase is a precursor of crystalline carbonated hydroxyapatite. It was found that the initially extracted amorphous mineral particles transformed into a crystalline mineral phase with time, and the proportion of crystalline mineral increased during bone maturation [19]. The transient ACP phase may conceivably be deposited directly inside the gap regions of collagen fibrils, but it may also be delivered as extrafibrillar particles [19]. This is consistent with the study that collagen mineralization via a transient ACP precursor phase *in vitro *to produce aligned intrafibrillar carbonated apatite crystals [20].

Several studies in different systems *in vivo *also have reported the presence of transient precursor calcium phosphate phases in the deposition of carbonated hydroxyapatite. Beniash performed a comprehensive analysis of the mineral phases in the early secretory enamel of the mandibular mouse incisors using four physical characterization methods. It was suggested that the outer, younger, early secretory enamel contained a transient disordered ACP phase, which transformed with time into the final apatitic crystalline mineral [17].

A variety of proteins and ions have been proposed to be involved in the biomineralization of ACP to HAP [21,22]. Dentin matrix protein1 (DMP1) is one of such biomineralization proteins [23]. In the report of He, it has been shown that two peptide motifs identified in DMP1 [motif-A (ESQES) and motif-B (QESQSEQDS)] enhanced in vitro HAP formation when immobilized on a glass substrate. It was demonstrated in another study that the synthesized artificial protein composed of these peptide motifs of DMP1 facilitated reorganization of the internal structure of amorphous particles into ordered crystalline states, i.e., the direct transformation of ACP to HAP, thereby acting as a nucleus for precipitation of crystalline calcium phosphate [24].

## 5. Transformation to Octacalcium Phosphate and Apatite

Studies on the preparation of hydroxyapatite [Ca_10_(PO_4_)_6_-(OH)_2_], the synthetic prototype of bone mineral, showed that the precipitation of initial solid phase from a calcium phosphate solution depends on the degree of its supersaturation [8]. A noncrystalline ACP precursor, approximating Ca_9_(PO_4_)_6 _in composition forms under conditions of high supersaturation [1,15]. This precursor ACP, unless stabilized in some way, transforms to thermodynamically more stable calcium phosphate phases or will be taken place by an autocatalytic solution-mediate crystallization process. On the other hand, the first solid to form in low supersaturated solutions is hydroxyapatite with Ca/P ratio of 1.67 obtained without precursor phases. Therefore, ACP is considered as a "mandatory precursor to apatite", and apatite can be formed in dilute solutions without going through this precursor [15]. The pH value also affects the initial solid phase in the precipitation of calcium and phosphate ions. Octacalcium phosphate (OCP) is the crystalline phase that initially forms when the reaction pH is less than 9.25, whereas apatite preferentially forms at higher pHs [25]. It is known that ACP is often the first-formed deposit i*n vitro*, at neutral pH and moderate supersaturation [26]. Transformation mechanism of ACP to apatite at physiological pH has been described as followings: firstly ACP dissolution, then a transient OCP solid phase reprecipitation through nucleation growth, and finally hydrolysis of the transient OCP phase into the thermodynamically more stable apatite by a topotactic reaction, which usually takes tens of hours [26].

Based on the analysis of the measured precipitate induction time and the structure of the developing solid phase, Feenstra proposed that OCP might be an intermediate in the conversion of ACP to apatitic calcium phosphate [27]. Since OCP or apatite crystals are generally found in association with ACP spherules, it is possible that ACP acts as a template for the growth of these crystal phases. Their formation, however, appears to take place by consuming ions largely supplied from the surrounding solution, rather than from direct hydrolysis of the solid amorphous material. At pH 10, transformation of ACP to poorly crystalline HAP may proceed without changes in the local calcium environment, but with the development of longer range order in the structure.

However, in contrast to these results at pH = 10, under physiological conditions the picture is quite different. Tung used a titration method to study the conversion of high-concentration ACP slurry to an apatite. There was a typical conversion kinetics clearly indicating two processes: the first process consumes acid, with the conversion of ACP to an OCP-like intermediary and the second process consumes base with the conversion of the OCP-like intermediate to apatite or, possibly, direct conversion of ACP to apatite. It was proposed that a stoichiometric HAP could be formed when there is no OCP-like intermediate phase, and a nonstoichiometric apatite product could be formed when an OCP-like intermediate phase is involved [28].

## 6. Biomedical and Dental Applications

ACP has been widely applied in biomedical field due to its excellent bioactivity, high cell adhesion, adjustable biodegradation rate and good osteoconduction [29-32]. As discussed above, the first quantitative studies on synthetic ACP were done in the mid 1960s [1]. From then on, more and more attention has been attracted in the development and the application of ACP-containing products, especially in orthopedic and dental fields. It is also used as filler in ionomer cements to fill carious lesions or as a colloidal suspension in toothpastes, chewing gums or mouthwashes to promote demineralization of carious lesions and/or to prevent tooth demineralization.

### 6.1 CPP-ACP

Casein phosphopeptides (CPP) contain the cluster sequence of -Ser (P)-Ser (P)-Ser (P)-Glu-Glu from casein [33,34]. Through these multiple phosphoseryl residues, CPP has a remarkable ability to stabilize clusters of ACP into CPP-ACP complexes, preventing their growth to the critical size required for nucleation, phase transformation and precipitation. In the United States, up to now, this product is primarily used for abrasive prophylaxis pastes and secondarily used for the treatment of tooth sensitivity especially after in-office bleaching procedures, ultrasonic scaling, hand scaling or root planing. However, its use for remineralizing dentin and enamel and preventing dental caries is an off-label application. Outside the United States, this product is marketed as GC Tooth Mousse [35,36].

**A **clinical trial of a mouthwash containing CPP-ACP showed that the contents of calcium and inorganic phosphate in supragingival plaque increased after use of the mouthwash for a three-day period [37]. Rose measured the affinity of Streptococcus mutans to CPP-ACP. It was demonstrated that CPP-ACP bound with about twice the affinity to the bacterial cells [38]. Hence, CPP-ACP binds well to plaque, providing a large calcium reservoir within plaque and slowing diffusion of free calcium. Additional evidence reported also by Rose indicates that CPP-ACP would compete with calcium for plaque Ca binding sites. As a result, this will reduce the amount of calcium bridging between the pellicle and adhering bacterial cells and between bacterial cells themselves [39]. This is likely to restrict mineral loss during a cariogenic episode and provide a potential source of calcium for the inhibition of demineralization and assist in subsequent remineralization.

A human *in situ *caries model has been used by Reynolds to study the ability of 1.0% CPP, 60-mM CaCl_2 _and 36-mM sodium phosphate, pH 7.0, solution to prevent enamel demineralization [40]. Two exposures of CPP-ACP solution *per *day to one side of the enamel slabs produced 51 ± 19% reduction in enamel mineral loss compared to the control side. Plaque exposed to CPP-ACP had 2.5 times more Ca and phosphorus than control plaque [36]. Reynolds also used an *in vitro *model system to study the effects of CPP-ACP solutions on remineralization of artificial lesions in human third molars. After a ten-day remineralization period, all solutions deposited mineral into the bodies of the lesions, with 1.0% CPP-ACP (pH 7.0) solution replacing 63.9 ± 20.1% of mineral lost at an averaged rate of 3.9 ± 0.8 × 10^-8 ^mol hydroxyapatite/m^2^/s. The remineralizing capacity was greater in the solutions with higher levels of CPP-stabilized free calcium and phosphate ions [41].

CPP-ACP and fluoride were shown to have additive effects in reducing caries experience [42]. Thus CPP-ACFP would add into the current fluoride-containing dentifrices as a toothpaste additive to improve the efficacy. Recent studies indicate that CPP-ACP can be incorporated into confectionery and drinks without adverse organoleptic effects [43]. CPP-ACP is a natural derivative of milk, therefore could have an important role as a food additive for the prevention of dental caries [44]. However, in 2008 Azarpazhooh systemically reviewed 98 articles on the clinical efficacy of casein derivatives and concluded that there was insufficient evidence (in quantity, quality or both) in existing clinical trials to make a recommendation regarding the long-term effectiveness of casein derivatives, specifically CPP-ACP, in preventing caries in vivo and treating dentin hypersensitivity or dry mouth [34].

### 6.2 ACP-filled polymeric composites

ACP has been evaluated as a filler phase in bioactive polymeric composites [45]. Skrtic has developed unique biologically active restorative materials containing ACP as filler encapsulated in a polymer binder, which may stimulate the repair of tooth structure because of releasing significant amounts of calcium and phosphate ions in a sustained manner [46-49]. In addition to excellent biocompatibility, the ACP-containing composites release calcium and phosphate ions into saliva milieus, especially in the oral environment caused by bacterial plaque or acidic foods. Then these ions can be deposited into tooth structures as apatitic mineral, which is similar to the hydroxyapatite found naturally in teeth and bone [50,51].

However, it was reported that the orthodontic ACP-containing adhesive showed lower bond strength. Dunn conducted an in vitro study to compare ACP-containing vs. conventional resin-based orthodontic adhesives [52]. Foster also compared the shear bond strength of orthodontic brackets using ACP-containing adhesive with a conventional adhesive and a resin-modified glass ionomer. In both studies, ACP-containing adhesive was demonstrated with lower, but clinically satisfactory bond strength as an orthodontic adhesive [53]. When comparing four new ACP-containing bonding systems, including Aegis Ortho, with a conventional bracket bonding system (Transbond XT), it was found that the traditional bonding systems achieved greater bond strengths than the newer ACP-containing ones. According to the study, however, Aegis Ortho had bond strengths sufficient for orthodontic use at 24-hour post-cure time. But the bracket might drift because of low viscosity of the material during laboratory bonding. The authors also found that Aegis Ortho had lower flexural strength, which would explain for the material failure at the adhesive-bracket interface rather than the enamel adhesive interface [54].

Compared with more commonly used silanated glass or ceramic filler, more hydrophilic and biodegradable ACP-filled composites exhibited inferior mechanical properties, durability and water sorption characteristics [55]. The uncontrolled aggregation of ACP particulates along with poor interfacial interaction plays a key role in adversely affecting their mechanical properties [56]. Their clinical applicability may be compromised by relatively poor filler/matrix interfacial adhesion and also by excessive water sorption that occurs in both resin and filler phases of these composites [42,46].

However, it has been demonstrated that it is possible to improve the remineralizing potential of ACP composites by introducing Si or Zr elements during low-temperature synthesis of the filler. Si- and Zr- ACPs enhanced the duration of mineral ion release through their ability to slow down the intra-composite ACP to HAP conversion [57]. It was also possible that non-ionic and anionic surfactants and poly (ethylene oxide) (PEO) introduced during the preparation of ACP play a role on the particle size distribution and compositional properties of ACP fillers [58]. The hydrophilic PEO is widely used in water compatible polymer systems because of its proven ability to undergo multiple hydrogen bonding interactions and stabilize cations by multiple chelation. The incorporated PEO in ACP fillers would also be expected to affect ACP's tendency to form aggregates and the water content of the ACP-containing composites. These properties would eventually affect both ion release kinetics and mechanical stability of composites [59]. It was found that surfactants introduced during the precipitation of ACP stabilized the amorphous solid phase against the conversion to apatite. The particle size of ACP was moderately reduced because of the introduction of anionic surfactant. Addition of PEO resulted in more pronounced ACP agglomeration but no changes of ACP's water content. Both surfactants and PEO lead to no changes in dry biaxial flexure strength of composites compared to the control Zr-ACP composites. However, their strength was drastically reduced in contrast to the control after prolonged exposure to aqueous milieu.

### 6.3 ACP in bone repair materials

Various compounds from calcium phosphate family have been extensively investigated as hard tissue repair materials due to their excellent biocompatibility [60]. It has been shown that the rate of new bone formation coincides more closely with the resorption rate of poorly crystalline apatites and ACP [61]. Additionally, ACP showed better osteoconductivity in vivo than apatite and its biodegradability was higher than that of tricalcium phosphate [25].

Clinically, it is widely accepted to use autograft and allograft materials to repair bone defects [29]. Recently, materials with ACP, hydroxyapatite and other calcium phosphate family members have been extensively investigated for alternative bone repair due to the limitations of traditional materials such as potential immunogenicity, insufficient supply and so on [62,63]. ACP and ACP/biopolymer composites have emerged as a new class of bone tissue engineering scaffold materials. Their excellent biocompatibility and osteoconductibility make them great materials for bone substitution and repair.

It has been shown that bone-like apatite materials have optimal surface characteristics for osteoblast cells to adhere, proliferate and differentiate, as a result, to favor bone formation and regeneration. An amorphous carbonated calcium phosphate ceramic was encapsulated within bioresorbable PLAGA microspheres and sintered to form a bioresorbable, highly porous, 3-dimensional scaffold. These noncrystalline and carbonated materials may be ideal for tissue ingrowth and potentially suitable for bone repair applications [64].

ACP was also incorporated into porous poly (L-lactic acid) (PLLA) to create a desired pore wall surface within bone tissue engineering scaffolds [65]. After being soaked in PBS, ACP aggregates in the composite experienced a fast and in situ transformation into bone-like apatite. The cell culture results also demonstrated that ACP/PLLA composite had an enhancement in cytocompatibility [65]. It has been demonstrated that ACP/PLLA material, which can experience morphological variations in the microstructure i**s **also supposed to be a suitable candidate as scaffold for cartilage tissue engineering [63,65].

## 7. Conclusions

ACP is usually formed as a metastable phase when calcium and phosphate ions in aqueous solution react to precipitate. The x-ray diffraction pattern, structure, morphology and infrared analysis results of ACP solids show typical noncrystalline characters within short-range order, instead of long-range periodic regularity. ACP act as an important intermediate product for in vitro and in vivo apatite formation. A variety of proteins and ions can increase the stability of ACP. ACP becomes increasingly significant in orthopedics and dentistry because of their excellent biocompatibility and mechanical properties. It is believed that ACP will be used even more extensively in the future due to due to the fast development of tissue engineering techniques and applied material science.

## Competing interests

The authors declare that they have no competing interests

## Authors' contributions

JZ, YL, WS and HZ have all been involved in drafting this review and have given final approval of the version to be published.
